# Genomic Stratification of Hematological Malignancies

**DOI:** 10.1097/HS9.0000000000000902

**Published:** 2023-05-25

**Authors:** Pauline Robbe, Anna Schuh

**Affiliations:** 1 RIKEN Center for Integrative Medical Sciences, Yokohama, Japan; 2 Department of Oncology, University of Oxford, United Kingdom

Hematological malignancies are a diverse group of blood cancers that arise from genetically altered bone marrow progenitors or secondary lymphoid cells that have escaped critical immune checkpoints. Next-generation sequencing (NGS) has increased our understanding of the genetic heterogeneity between and within hematological malignancies. The specific types of genetic alterations mostly studied in the context of precision medicine and companion diagnostics include somatic small mutations and structural variants (SVs) such as copy-number alterations (CNAs) and gene fusions.

Robust evidence of the clinical utility of molecular classifications has been generated for several hematological malignancies and is reflected in the fifth edition of the World Health Organization (WHO) classification.^1,2^ Genetic alterations used for classification were mainly discovered using conventional diagnostics or targeted methodologies such as fluorescence in situ hybridization (FISH), microarrays, targeted NGS panels, and labor intensive/technically challenging methods such as conventional karyotyping, requiring culturing fresh cells, that often lacks resolution, misses alterations, and presents high failure rates.^3–5^ More recently, approaches such as RNA sequencing (RNA-seq) and whole exome/genome sequencing (WES/WGS) are becoming available in the diagnostic laboratories. The most comprehensive approach, WGS, has been shown to provide equivalent if not superior information in a single assay revealing additional cryptic changes and other alterations missed by conventional testing. However, WGS comes with significant challenges of increased analytical requirements and clinical interpretation. These were extensively reviewed in a series of dedicated publications.^6–8^

Here, we are summarizing recent advances in clinical applications of genomics for diagnosis and risk-stratification of hematological malignancies (Table 1). We are focusing on how the adoption of integrated genomics, including WGS as a single-test modality, could transform clinical practice and refine precision medicine.

**Table 1:** Genomic Stratification of Hematological Malignancies

Malignancy	Reference	Methods	Genetic Markers	n Patients	Main Findings
AML	Patel et al. N Engl J Med 2012; 366:1079–1089.	Sanger sequencing	Mutations in 18 genes	398 (+validation in 104)	▪ Reclassification of 26% of intermediate-risk patients in a favorable group and 39% in an unfavorable group ▪ Identification of prognostic genes ▪ Improvemen of risk-stratification
AML	Papaemmanuil et al. N Engl J Med 2016;374:2209–2221	Targeted NGS	Mutations in 111 cancer genes Copy-number alterations Fusion genes	1540	▪ Definition of 14 distinct (including 11 molecularly-defined) subgroups ▪ Increase from 48% to 80% the proportion of patients classified in 1 distinct subgroup ▪ Improved OS prediction compared with the ELN classification: 71% of patients with an accurate prediction compared with 64%
MDS	Bersanelli et al. J Clin Oncol 2021;39:1223–1233	Targeted NGS	Cytogenetic data, mutations in 47 cancer genes	2043	▪ Definition of 8 genomically-defined subgroups ▪ Improvement of the concordance of the observed outcomes in the EuroMDS cohort when integrating genomic data 0.742 (training cohort) and 0.709 (testing cohort), compared with the IPSS-R 0.576 (training cohort) and 0.567 (testing cohort)
MDS	Nazha et al. J Clin Oncol 2021;39:3737–3746	Targeted NGS	Clinical data, cytogenetic data, mutations in 38 genes	1,471	▪ Improvement of the c-index scores reflecting cross-validation when using their model compared with the IPSS-R in predicting OS (0.72–0.74 vs 0.67) and as well as leukemia transformation
MDS	Bernard et al. NEJM Evid 1:2022, 2022		Cytogenetic data, mutations in 152 genes	2957	▪ Development of the model the IPSS-M, which improved on the IPSS-R ▪ Improvement of the predictions for leukaemia-free survival, OS, and AML transformation ▪ Reclassification of 46% of patients, of which 74% were upstaged
ALL	Roberts et al. N Engl J Med 2014;371:1005–1015	RNA-seq	Gene rearrangements, gene fusions, and mutations	1725	▪ Identification of several subgroups of Ph-like B-ALL
ALL	Paietta E et al. Blood. 2021 Sep 16;138(11):948–958.	RNA-seq, microarrays, PCR NGS panel and Sanger Sequencing	Chromosomal and arm level CNAs, known fusions, expression profiles, gene mutations	264	▪ Definition of 11 subgroups ▪ A comprehensive molecular classification in adult BCR-ABL1-negative B-ALL could dramatically improve the clinical trial risk classification (currently: age >35 y or WBC count >30 × 109/L) ▪ 89% of patients were assigned one of the subtypes
ALL	Li et al. Proc Natl Acad Sci USA 2018;115:E11711–E11720.	RNA-seq	Expression profiles, gene fusions, and gene mutations	1223	▪ Definition of 14 molecular subgroups including 6 novel ones with distinct clinical outcomes effectively assigning a subgroup to an additional 15% of patients.
ALL	Gu et al. Nat Genet. 2019 Feb;51(2):296–307.	RNA-seq	Chromosomal rearrangements, sequence mutations, and genomic alterations	1988	▪ Definition of 23 subgroups ▪ Increase from 76% to 94% the patients with a defined subtype linked to specific clinical outcome
ALL	Brady et al. Nat Genet. 2022;54:1376–1389.	RNA-seq, WES, WGS	Expression profiles, chromosomal rearrangements, gene mutations and copy-number alteration	2754	▪ Nearly all patients carried at least 1 putative driver gene alteration detectable by NGS ▪ The prognosis of some of these subgroups can be further refined thanks to the presence/absence of these secondary genetic alterations. For example, they found that the presence of somatic mutations in *SETD2* in hyperdiploid ALL was associated with a reduced OS (5-y EFS 46.9% vs 94.9%).
Lymphoma	Reddy et al. Cell. 2017;171 (2):481–494.e15	WES and RNA-seq	150 mutated drivers and gene expression markers (COO, MYC, and BCL2)	1001	▪ Definition of a predictive model for survival using a multivariate supervised learning approach including 150 mutated drivers and gene expression markers (COO, MYC, and BCL2) alone or in combination (if recurrently co-occuring in >20 patients) totaling 313 possible combinations. ▪ Integrated genomic models performed better than simplified models such as DNA-only, RNA-only, or DNA-RNA when taking variables individually only. ▪ The integrated genomic risk model outperformed the known classification discriminating low-risk and high-risk cases.
Lymphoma	Chapuy et al. Nat Med. 2018;24:679–690.	WES with an expanded bait set to capture known SVs	158 mutated driver genes, CNAs and SVs	304	▪ Harvard classification ▪ establishment of 5 subgroups ▪ Two subgroups, cluster 5 and 1, were of ABC-types, represented 40% of the cohort, had distinct genomic landscapes, which could be treated by different targeted strategies. Cluster 3 and 4 were GCB type and also presented different mutation patterns suggesting different potential therapeutic strategies. Cluster 2 was characterized by bi-allelic inactivation of TP53. Finally, 4% of patients were defined by the absence of the drivers included in the model (cluster 0). ▪ These subgroups presented different associations to progression-free survival and retained significance in a multivariate analysis including IPI.
Lymphoma	Wright et al. Cancer Cell 2020;37:551–568.e14	Whole-exome data	Driver mutations, CNAs, and known rearrangement	574	▪ LymphGen algorithm ▪ A single tumor (1) can match a subtype without carrying the genetic hallmarks and/or (2) can match several genetic subtypes, named “genetically composite.” ▪ Classification prediction used driver mutation and known rearrangement data and fit the patients into 6 predetermined subgroups: 4 based on the prior knowledge MCD (MYD88 L265P and CD79B mutations), BN2 (BCL6 translocations and NOTCH2 mutations), N1 (NOTCH1 mutations), and EZB (EZH2 mutations and BCL2 translocations); and 2 based on recurrent alterations in their cohort: “A53” (aneuploid with TP53 inactivation) and “ST2” (SGK1 and TET2 mutations). ▪ The algorithm classified 63.1% of cases, which is 9.1% more than by simply classifying cases based on the genetic hallmarks of the 6 subgroups. ▪ Although the OS of each subtype across datasets was not exactly reproducible, they were visually similar on Kaplan-Meier analyses.
Lymphoma	Lacy et al. Blood 135, 1759–1771 (2020)	Targeted NGS panel	Driver mutations, known CNAs	928	▪ HMRN classifier. ▪ Detected 5 molecular subtypes, namely MYD88, BCL2, SOCS1/SGK1, TET2/SGK1, and NOTCH2. Three subgroups, BCL2, NOTCH2, and MYD88, validated previous findings in prognosis prediction. The other 2 subgroups, SOCS1/SGK1 and TET2/SGK1, were newly defined and associated with excellent prognosis.
CLL	Rossi et al. Blood. 2013;121:1403–1412.	Sanger sequencing and cytogenetic analysis	Driver mutations, CNAs		▪ Integrating mutational and cytogenetic analysis. ▪ Recursive partitioning making a decision tree included TP53 disruption, BIRC3 disruption, SF3B1 mutation, NOTCH1 mutation, Del(11q)
CLL	Taus et al. Front Genet. 2021;12.	WES	Driver mutations grouped in pathways	506	▪ Reanalysis of the ICGC dataset previously published, including samples from 506 CLL patients. ▪ Defining 4 clusters associated predictor of TTFT, independently of IGHV mutational status.
CLL	Knisbacher et al. Nat Genet. 2022;54:1664–1674.	RNA-seq	Expression profiles	603	▪ Identification of 8 ECs ▪ ECs were highly correlated with IGHV mutational status and/or epitype: EC-u1 and EC-u2 were associated with unmutated IGHV/ naive-like CLL; EC-m1, EC-m2, EC-m3, and EC-m4 were associated with mutated IGHV/memory-like CLL; EC-i was associated with intermediate CLL; and EC-o was not associated with any particular subtype. ▪ EC-i was associated with adverse FFS whereas EC-o, EC-m3, and EC-m4 with good outcome
CLL	Robbe et al. Nat Genet, 2022;54:1675–1689.	WGS	Driver mutations, noncoding mutations, CNAs, SVs, mutational signatures, pathways, telomere lengths, other genome-wide measures.	485	▪ Integration of all types of variants in coding and noncoding regions using WGS in 485 patients enrolled in clinical trials. ▪ Defining 5 genomically distinct subgroups using 186 genomic features per genome ▪ Subgroups present distinct responses to treatment. ▪ WGS defined subgroups cannot be recapitulated with only known CLL markers.

AML = acute myeloid leukemia; B-ALL = B-cell acute lymphoblastic leukemia; CLL = chronic lymphocytic leukemia; CNA = copy-number alteration; COO = cell-of-origin; EC = expression clusters; EFS = Event Free Survival; ELN = European Leukemia Network; FFS = failure-free survival; GCB = germinal center B-cell; HMRN = Haematological Malignancy Research Network; ICGC = International Cancer Genome Consortium; IGHV = immunoglobulin heavy chain variable; IPI = International Prognostic Index; IPSS-M = the Molecular International Prognostic Scoring System; IPSS-R = Revised International Prognostic Scoring System; MCD = MYD88 / CD79B mutation subgroup; NGS = Next-generation sequencing; OS = overall survival; PCR = polymerase chain reaction; RNA-seq = RNA sequencing; SVs = structural variants; TTFT = time to first treatment; WBC = white blood cell; WES/WGS = whole exome/genome sequencing.

## MYELOID MALIGNANCIES

For decades, conventional karyotyping combined with an ever-increasing panel of FISH probes has been a cornerstone in the diagnostic work-up of patients with myeloid malignancies. Routine testing detects CNAs, gene fusions, and other gene rearrangements, but cannot reveal somatic SNVs/indels^9–13^ that also contribute to refining molecular classifications of acute myeloid leukemia (AML)^14,15^ and myelodysplastic neoplasms (MDS).^16–19^

In AML, 2 key studies have proposed genomic stratification models^14,15^ (Table 1). Patel et al found that adding gene mutation data improved the cytogenetically defined prognostic classification so that less patients were assigned to the intermediate prognostic group and more to the favorable group (26%) or to unfavorable group (39%). Papaemmanuil and colleagues proposed a molecularly driven subgrouping integrating mutations in 111 cancer genes, CNAs, and fusion genes, together with other typical characteristics such as clinical variables. They defined 14 subgroups increasing from 48% to 80% the proportion of patients classified in 1 distinct subgroup. Besides, their classification improved overall survival prediction compared with the 2017 European Leukemia Network (ELN) classification^20^: 71% of patients with an accurate prediction compared with 64%.

In MDS, 3 studies proposed improved classifications by integrating driver mutations with clinical data and cytogenetics^21–23^ (Table 1). By screening for acquired SNVs/indels using a panel of 47 known driver genes, Bersanelli and colleagues defined 8 subgroups that are mutually exclusive to each other.^21^ The integration of genomic data improved the classification compared with the Revised International Prognostic Scoring System (IPSS-R). Nazha and colleagues proposed a model integrating mutations in 7 drivers as well as the number of mutated drivers with other clinical data.^22^ Subsequently, the Molecular International Prognostic Scoring System (IPSS-M), adding mutations data from 152 MDS-related genes, improved predictions for leukemia-free survival, overall survival, and AML transformation compared with the IPSS-R.^23^ Totally 46% of patients were reclassified, of which 74% were upstaged. This classification was recently validated in an independent cohort of 2876 patients, although the proportion of upstaged patients was only half in this cohort.^24^

As a result, the 2017 ELN and the 2022 WHO classifications for myeloid malignancies now include key driver mutations. A new subgroup in the WHO classification named “AML with other defined genetic alterations,” allows for the reporting of additional molecular findings of potential clinical significance, highlighting the importance of comprehensive screening for all different types of mutations and their interactions across the genome. To address the clinical need for comprehensive molecular diagnostics of AML/MDS, WGS is increasingly being evaluated as a clinical tool in this indication.

Accurate WGS analysis of tumor samples requires the sequencing of both tumor and germline DNA in order to identify the somatic changes. One of the major limitations of using WGS for AML/MDS risk-stratification has been the contamination of matched germline samples with tumor DNA. Obtaining tumor-free germline DNA remains challenging as peripheral blood, used in solid tumors as a source of germline DNA, cannot be used. Saliva or buccal swabs of patients with AML/MDS are also often contaminated with tumor DNA.^25–27^ The same applies to sorted and cultured T cells. Only cultured fibroblasts obtained from skin biopsies at the time of bone marrow examination are thought to be relatively tumor-free. Recent advances in computational processing might mitigate this challenge by accurately assessing the tumor in normal contamination^27^ and improving detection.^28^ Besides, diagnostic WGS could be implemented without the requirement for paired germline as recently investigated for AML and MDS.^29^ The study showed WGS was superior in detecting all CNAs and SVs reported by conventional methods and even detecting additional ones for 25% of patients. These SVs were missed or could not be resolved by conventional testing, or conventional testing failed. They proposed a tier-levelled reporting of SVs, listing high-quality but less recurrent SV and novel ones affecting driver genes. For small mutations, to mitigate the lack of germline samples, an in-silico panel was applied by screening only mutations in genes of interest. Although some low-level subclonal variants were missed by WGS, the positive predictive value of clinically actionable drivers was 99%. Importantly, this study showed a favorable turnaround time for WGS (5.1 days) compared with conventional cytogenetics (10 days) and overall cost was like those of current tests. Other efforts have been evaluating the implementation of WGS in combination with whole transcriptomic sequencing to detect all types of clinically relevant alterations.^30,31^

## ACUTE LYMPHOBLASTIC LEUKEMIA

Similar to AML/MDS, acute lymphoblastic leukemia presents a wide variety of genomic alterations associated with distinct subgroups mostly determined by the presence of (1) chromosomal aneuploidies, (2) gene fusions, and (3) other chromosomal rearrangements such as the intrachromosomal amplification of chromosome 21, which can be detected by conventional karyotyping combined with FISH.^32,33^ More recently, optical genome mapping has been introduced, which provides digital karyotypes, without the need to culture the leukemia cells.^34^

As patients who do not fit in any known subgroup cannot benefit from risk-adapted therapy or targeted therapies, efforts have led to refining existing subgroups and discovering additional ones especially defined by (1) cryptic rearrangements and fusions, (2) rearrangements involving several partners converging toward the same gene, (3) expression profiles, and (4) single-point mutations^35^ (Table 1). By integrating several methods including RNA-seq, Paietta et al defined distinct subtypes classifying 89% of patients.^36^ Other RNA-seq based classification emerged (1) resolving additional recurrent rearrangements, (2) defining novel expression profiles (phenocopy subtypes), and (3) finding recurrent mutated genes, notably *PAX5* mutations.^37,38^ Brady and colleagues applied WGS, WES, and transcriptome sequencing, leveraging both information from previous RNA-seq classification and further refining the subgroups based on the presence of secondary genetic alterations.^39^

Collectively, these studies ask the important question of the choice of methodology to use in clinical practice to comprehensively identify a growing number of molecular subtypes defined by both (1) genomic alterations including mutations, CNAs, and structural rearrangements, some involving noncoding regions; and (2) expression profiles. Recent approaches include the use of bespoke targeted NGS panels^40^ or WGS as a stand-alone test.^41^ The latter demonstrated the potential of WGS to resolve clinically relevant subgroups, in particular cryptic rearrangements and proposed a novel classification based on the comprehensive analysis of somatic mutations and SVs.

## LYMPHOMAS

The classification of diffuse large B-cell lymphoma, the most common aggressive lymphoma, is complex and includes the detection of (1) protein expression, (2) gene expression profiles, (3) translocations, and (4) CNAs.^2^ Multiple genomic studies highlighted the heterogeneous molecular landscape and presence of recurrently mutated genes.^42–47^ More recently, 4 different groups independently proposed new prognostic classifications using integrated genomic approaches supported by known markers: (1) cell-of-origin (COO), (2) double-hit signature/molecular high-grade defined by RNA-seq, and (3) double-hit lymphoma/triple-hit lymphoma (DHL/THL) defined by translocations^47–52^ (Table 1). The first study found that integrated genomic models including both DNA and RNA data as well as combinations of mutations performed better than simplified models such as DNA-only or RNA-only or without combinations. This model identified different low-risk and high-risk disease in subgroups divided by COO, DHL, and the International Prognostic Index (IPI), demonstrating its complementarity to these known markers and superior classification.^47^ The Harvard study used WES with an expanded bait set to capture known SVs and classified successfully 96% of the patients. As the DHL/THL were not confined to a single subgroup of their new classification, the author suggested that the currently established designations are not sufficiently precise to fully capture the molecular basis of the disease. These subgroups presented different associations to progression-free survival and retained significance in a multivariate analysis including IPI.^48^ Wright and colleagues developed a probabilistic classification named the LymphGen based on the predetermined 6 subgroups carrying one of the genetic hallmark alterations.^52^ The novelty of this algorithm consisted in allowing a single tumor to (1) match a subtype without carrying the genetic hallmarks, and/or to (2) match several genetic subtypes, named genetically composite. They successfully classified 63% of cases which is 9% more than by simply classifying cases based on the genetic hallmarks of the 6 subgroups. The LymphGen algorithm was adapted to work from various input types and validated using the datasets from the 2 previously cited studies.^48,50^ As in previous work, known markers did not all fit into single genetically defined subgroups, highlighting the complementarity between the different classifications. Finally, Lacy et al proposed another classification using a targeted NGS panel identifying driver mutations and selected CNAs, named the Haematological Malignancy Research Network (HMRN) classifier.^51^ Using unsupervised clustering they defined 5 subgroups Although this classification failed to delineate key subgroups identified by LymphGen, some were subsequently added in a follow-up study using supervised clustering.^53^

Overall, despite using different sequencing technologies, targets, statistical approaches, and clustering methods, some consensus can be noted between these clustering methods, proving the robustness of these new subgroups. A detailed comparison between subgroups of the different classification has been previously published.^54^ Differences remaining could be caused by differences in cohort sizes as some subgroups are quite rare and/or clustering approaches (supervised versus unsupervised). In addition, the highest variability came from the number of cases classified by each model: the Harvard model classified 96% of patients, whereas LymphGen and HMRN classified 63% and 73%, respectively. In the absence of an existing gold standard and validation data demonstrating clinical utility from clinical trials, these differences raise the question of which methodology to favor.

In addition to the lack of full consensus, many challenges must be overcome before these approaches can be applied in routine diagnostics: standardization of laboratory methods, bioinformatics and statistical approaches, and availability of computational methods to overcome FFPE artifacts.^55,56^ Finally, the different emerging methodologies for analyzing circulating lymphoma DNA from plasma and their potential role in diagnosis, risk-stratification, and response monitoring of lymphomas must be fully evaluated.

## CHRONIC LYMPHOCYTIC LEUKEMIA

For decades, prognosis and stratification in chronic lymphocytic leukemia (CLL) have been largely relying on a combination of clinical examination,^57,58^ immunoglobulin heavy chain variable (IGHV) mutation status, and the presence or absence of *TP53* disruption.^56,57^ To predict time to first treatment of early-stage CLL, 2 classification systems have been proposed: the International Prognostic Score for early-stage CLL/SLL^59^ and the CLL1 prognostic model.^60^ For outcome prediction following treatment, the Four Factor Prognostic Model^61^ and the CLL International Prognostic Index^62^ consider both clinical information, *TP53* disruption (mutation/CNA), and IGHV mutational status. In addition to *TP53* disruption and IGHV mutational status, the 2022 WHO classification recommended additional markers for comprehensive prognostication of CLL, including del(11q), del(13q), and trisomy 12; B-cell receptor stereotype subset analysis (subset 2 configuration); complex karyotype and *BTK*, *PLCG2*, and *BCL2* mutation analysis in the context of receiving targeted therapy.^2^

In the past decade, multiple studies using combinations of mutational and cytogenetic analyses or WES/WGS have discovered a long list of putative driver genes, recurrent CNAs, the presence of complex karyotypes and recurrent combinations of drivers.^63–75^ The genomic landscape of CLL is highly heterogeneous and the scarcity of recurrent driver genes across CLL cohorts has prevented extensive validation and inclusion of such novel markers in updated risk-stratification models. Several attempts at genetically driven classifications that overcome this challenge have been made (Table 1). Rossi and colleagues proposed recursive partitioning, a decision tree that included *TP53* disruption, *BIRC3* disruption, *SF3B1* mutation, *NOTCH1* mutation, Del(11q)^76^ to hierarchically order genetic information. Others proposed a pathway-based stratification of CLL patients using mutation data to reduce the mutational heterogeneity across patients.^77^

Overall, most other previous studies focused on identifying independent prognostic and predictive genetic markers often using multivariable Cox regression models. However, such approaches lack the ability to model complex relationships between markers and outcome. Therefore, to go beyond single-marker and regression model approaches, 2 recent studies have explored risk-stratification methodologies that integrate all genomic variables into a risk model applying machine learning algorithms.^78,79^ Knisbacher and colleagues applied unsupervised clustering using RNA-seq data. They identified 8 expression clusters highly correlated with IGHV mutational status and/or epitype. In multivariate analysis including genetic alterations, epitypes, epiCMIT, and expression clusters, the authors showed the prognostic impact of the expression clusters on failure-free survival and overall survival. Complementary to this study, our group highlighted the potential of WGS to inform future risk-stratification for treatment decisions. Although many genomic studies in hematological malignancies have been performed in recent years, most of them either used multiple different targeted laboratory tests to associate genomics with clinical outcome or employed multiomics with WGS but focused on a limited number of types of acquired mutations without integrating them into a risk model (Table 1). By contrast, we fully integrated 186 different features derived from a single-genomic test including coding and noncoding variants, CNAs, SVs, and genome-wide features, such as telomere length, mutational signatures, and genomic complexity, among others. We identified 5 genomic subgroups: 3 associated with unmutated IGHV status and 2 associated with mutated IGHV status. We showed that the subgroups have distinct progression-free survival in a cohort of patients receiving chemoimmunotherapy. Importantly, by using solely known coding drivers and recurrent CNAs, these subgroups could not be recapitulated, implying that they are also defined by other genomic changes beyond the known coding drivers and CNAs. These findings demonstrate the additional power offered by genome-wide analyses.

Both studies provide important insights into the application of genome-wide/integrated genomics for the subgrouping of CLL. Both studies showed that the clusters are associated with distinct genomic events, yet not carried by all patients. Also, both studies seem to have identified similar subgroups, for instance subgroups with a high proportion of cases with del(11q) or trisomy 12 alterations and other overlapping features. Further studies will be required to formally compare these 2 new classifications and assess them in the context of risk-stratification of early-stage CLL and treatment with targeted therapies. Importantly, limiting the clinical investigation to single genes/panel of genes and grouping patients based on the presence/absence of these alterations cannot fully capture the integrality of genomic, epigenomic, and transcriptomic changes occurring in CLL.

Finally, given the complexity of the CLL genome and the growing evidence that genome-wide screening is required, Klintman et al have proposed a validation of the use of WGS in the clinic for CLL.^80^ In their study, the authors showed a high concordance between WGS and other established methods, FISH, SNP array, targeted NGS in a cohort of 64 patients. The clinically relevant genetic alterations were validated including Del(17p), del(11q), del(13q), trisomy 12, *TP53* mutations, and complex karyotypes. As the majority of discrepancies in this study were resolved after visual inspection, it is expected that the current high-pace advances of computational analysis will solve this challenge. With the increased number of targeted therapy available, emergence of new driver genes and genome-wide genetic markers, this study demonstrated the feasibility of WGS as a single genetic test in a clinical setting.

## CONCLUSION AND PERSPECTIVE

The use of routine integrated NGS testing for precision medicine of hematological malignancies presents a promising opportunity to improve patient outcomes by enabling more accurate diagnosis, more personalized treatment, and better understanding of disease biology. To date, several classifications using these approaches have been proposed. However, realizing the full potential of these technologies will require collaboration between academic and industry researchers, clinicians, and patients to address the current lack of independent clinical validation and utility data.

The majority of genomic stratification models presented here (Table 1) requires several tests to derive the most complete classification. We argue that most if not all hematological malignancies could be classified using WGS alone as it can resolve all coding and noncoding small mutations, CNAs, mutational burden, the presence of complex karyotypes, gene fusions, and cryptic rearrangements. With the continuous reduction of cost and analytical burden, we envisage WGS to become a powerful clinical tool for the management of blood cancers.

## AUTHOR CONTRIBUTIONS

PR and AS conceptualized, wrote, and finalized the article.

## DISCLOSURES

In the past 5 years, AS has received in-kind contributions from Illumina and Oxford Nanopore Technology and is a shareholder of Illumina. She is a company director and shareholder of SERENOx Ltd. AS has received honoraria from Exact Sciences, Janssen, Astra Zeneca, AbbVie and Beigene, nonrestricted research grants from Janssen and Astra Zeneca and an educational grant from AbbVie. The other author has no conflicts of interest to disclose.

## SOURCES OF FUNDING

The work of PR was supported by an institutional funding provided by the Japanese Ministry of Education, Culture, Sports, Science and Technology to RIKEN Center for Integrative Medical Sciences. The work of AS was supported by the Department of Health’s National Institute for Health Research Biomedical Research Centers funding scheme. The views expressed in this publication are those of the authors and not necessarily those of the Department of Health.
